# Current Understanding of Meningitis Pathogens, Immunity Gap, and Future Prevention Strategies in Sri Lanka: A Narrative Review

**DOI:** 10.1155/ijm/6433600

**Published:** 2026-08-17

**Authors:** Thashmila Heshani, Dilshan Sandaru, Sachindu Madhushara, Sarfaraz Ahmad Ejazi, Bhagya Deepachandi

**Affiliations:** ^1^ Department of Life Sciences, Faculty of Science, NSBM Green University, Homagama, Sri Lanka; ^2^ Department of Health Sciences, Faculty of Science, NSBM Green University, Homagama, Sri Lanka; ^3^ Fischell Department of Bioengineering, A. James Clark School of Engineering, University of Maryland, College Park, Maryland, USA, umaryland.edu

**Keywords:** meningitis, molecular diagnostics, Sri Lanka, vaccination gap, WHO roadmap

## Abstract

Meningitis, an acute and life‐threatening inflammation of the protective membranes covering the brain and spinal cord caused primarily by bacterial, viral, fungal, or parasitic infections, remains a major global health challenge, causing significant morbidity and mortality, particularly among children and vulnerable populations. The Sri Lankan epidemiological unit reported approximately 1000–1500 meningitis cases annually. This review summarizes current knowledge on meningitis in Sri Lanka, drawing on global evidence and locally published studies to examine epidemiology, clinical presentation, diagnostic approaches, immunity gap, and future priorities. Most studies indicate that bacterial meningitis, particularly caused by *Streptococcus pneumoniae*, *Neisseria meningitidis*, and *Haemophilus influenzae*, remains the most severe and clinically significant form. Viral etiologies, especially enteroviruses, are also commonly reported. Although the introduction of the *H. influenzae* Type b (Hib) vaccine has significantly reduced disease burden, important immunity gaps persist due to limited pneumococcus serotype coverage and the absence of routine meningococcal vaccination. Diagnostic challenges, including delays in cerebrospinal fluid collection, limited access to molecular testing, and preanalytical errors, hinder early detection and accurate pathogen identification. Future directions should focus on strengthening molecular diagnostic capacity, strengthening point‐of‐care rapid antigen assays and decentralized molecular diagnostics, expanding serotype surveillance, improving sample collection practices, and evaluating the feasibility of introducing meningococcal vaccination into the national immunization schedule. Bridging these gaps is essential for aligning Sri Lanka′s efforts with global targets outlined in the World Health Organization′s (WHO) Defeating Meningitis by 2030 roadmap.

## 1. Introduction

Meningitis remains a major global public health concern which is defined as the inflammation of the protective membranes (meninges) surrounding the brain and spinal cord, triggered predominantly by the invasion of infectious pathogens into the central nervous system (CNS), though occasionally resulting from non‐infectious triggers [1, 2]. The condition carries a high risk of mortality and long‐term sequelae, necessitating immediate medical attention. Infectious meningitis is caused by various infectious agents, including bacteria, viruses, fungi, and parasites [3, 4]. A smaller proportion of cases are linked to non‐infectious factors such as autoimmune disorders, malignancies, or certain medications. The WHO has identified meningitis as a leading infectious cause of death and disability, capable of triggering public health emergencies. In response, the WHO [5] introduced the Defeating Meningitis by 2030 global roadmap, which establishes an overarching vision “toward a world free of meningitis.” This strategic plan is aimed at reducing deaths from bacterial meningitis by 70%, halving the number of vaccine‐preventable bacterial meningitis cases, and eliminating recurrent epidemics by 2030 [6].

In Sri Lanka, as worldwide, bacterial meningitis represents the most severe form of the disease, involving acute infection of the CNS [7, 8]. It is characterized by rapid progression and a significant risk of long‐term complications, including hearing loss, neurological defects, and cognitive impairment [9]. Vaccination remains the cornerstone of prevention for bacterial meningitis [10]. For timely and accurate diagnosis, molecular techniques such as multiplex polymerase chain reaction (PCR) are considered highly effective in identifying causative pathogens [11].

This literature review synthesizes the current state of knowledge on meningitis with a particular focus on Sri Lanka. It examines key areas, including global and local epidemiology, clinical presentation, risk factors, diagnostic approaches, and the current management strategies within the country. The review also assesses the status of Sri Lanka′s national vaccination programs, analyzing their strengths and limitations to identify persistent immunity gaps related to vaccination coverage, serotype/serogroup distribution, and surveillance. Furthermore, it highlights the critical role of early diagnosis and outlines future directions for research and public health action. The primary focus of this review is on bacterial and viral etiologies of meningitis.

## 2. Search Strategy and Selection Criteria

A comprehensive literature search was conducted in databases such as PubMed, Google Scholar, and the Sri Lanka Journals Online (SLJOL) portal for relevant studies published up to May 2026. Search terms included combinations of keywords such as “meningitis,” “Sri Lanka,” “epidemiology,” “bacterial meningitis,” “viral meningitis,” “molecular diagnosis,” and “vaccination.” The inclusion criteria were peer‐reviewed articles, case series, and official reports released by the Epidemiological Unit of the Ministry of Health, Sri Lanka, on the prevalence of pathogens, clinical characteristics, or diagnostic gaps in Sri Lanka. Non‐English publications and studies that exclusively focused on non‐infectious disease of the CNS were excluded.

## 3. Global Overview of Meningitis

Worldwide, the greatest burden of meningitis is found in sub‐Saharan Africa, an area known as the “African meningitis belt” that extends from Senegal to Ethiopia [12]. This region is recognized as one of the most severely affected by meningococcal meningitis [4]. Meningitis can affect individuals of all ages across diverse regions, with causative pathogens varying according to age, immune status, and environmental exposure. Newborns are particularly susceptible to Group B *Streptococcus agalactiae*, *Escherichia coli*, and *Listeria monocytogenes*, whereas children and adolescents face higher risks from *Neisseria meningitidis*, *Streptococcus pneumoniae*, and *Haemophilus influenzae* [2]. In adults, pneumococcus and meningococcus remain the predominant causes of bacterial meningitis [13]. Furthermore, individuals with weakened immune systems, especially those living with human immunodeficiency virus (HIV), face a greater risk of meningitis. Because their natural defenses are compromised, they are vulnerable to a wider range of organisms that can cause the disease, making prevention and timely treatment even more critical [6].

Globally, an estimated 2.51 million cases of meningitis were reported in 2019, including approximately 1.6 million cases of bacterial meningitis [14]. The disease accounted for nearly 236,000 deaths worldwide during the same year, highlighting its continued impact despite advancements in antimicrobial therapy and immunization strategies [14]. The WHO global roadmap sets out ambitious but practical goals. It calls for ending bacterial meningitis epidemics, cutting vaccine‐preventable cases in half, and reducing deaths by 70%. Beyond prevention, the plan also emphasizes strengthening clinical care and rehabilitation, ensuring that patients not only survive but also receive the support they need to recover fully [5].

Efforts to control meningitis increasingly emphasize prevention through vaccination. Immunization against *H. influenzae* Type b (Hib) remains the most established approach, offering reliable and cost‐effective protection through combination vaccines [15]. Pneumococcal conjugate vaccines (PCVs) have also become more accessible, with falling costs encouraging wider uptake [15]. Despite these advances, countries lacking donor support still face challenges related to affordability and access. Furthermore, current vaccine formulations do not provide full coverage against all circulating pneumococcal serotypes [16]. Global burden frameworks identify broad hyperendemic belts across sub‐Saharan Africa; however, South Asian settings show distinct epidemiological patterns driven by dense urbanization, changing regional carriage patterns, and varying healthcare infrastructure [12]. An evaluation of the dynamics of a neighboring country, such as the progressive rollout of the PCV in India to address its high continental burden, provides an essential understanding of intermediate disease transmission across shared geographic zones [17, 18].

## 4. Epidemiology of Meningitis in Sri Lanka

**Figure 1:**
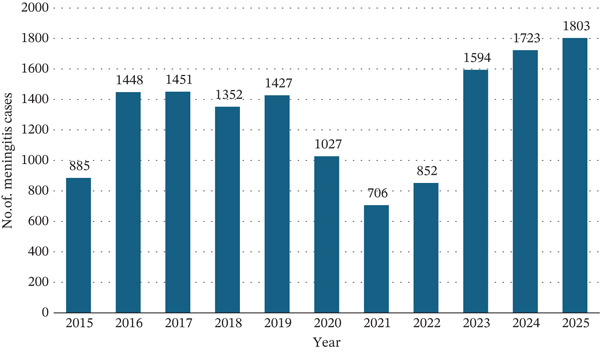
Annual meningitis cases reported in Sri Lanka from 2015 to 2025 [19]. Source: Author, created using data from the Epidemiology Unit, Sri Lanka.

Meningitis remains a significant public health concern in Sri Lanka and continues to be a notifiable condition under the national surveillance system [19]. According to the Epidemiology Unit, approximately 1000–1500 meningitis cases are reported annually in the country [20]. The annual distribution of meningitis cases in Sri Lanka from 2015 to October 2025 is shown in Figure 1.

The data presented in Figure 1 indicate a rising trend in meningitis incidence in Sri Lanka over the past decade. The years 2023, 2024, and 2025 represent peaks in case numbers, marking the highest reported incidence within the period shown [19].

**Figure 2:**
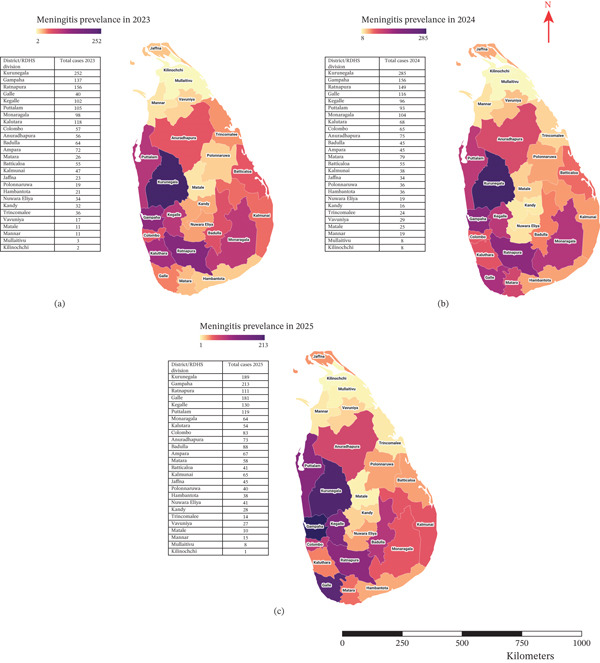
Geographical distribution and case intensity of reported meningitis across Sri Lankan RDHS divisions [19]. Panels show annual prevalence for (a) 2023, (b) 2024, and (c) 2025. The color gradient ranges from light yellow/orange (low incidence) to deep purple (high incidence), tracking shifts in localized disease burdens. Source: Author, created with datawrapper.de.

Figure 2 shows a geographical heatmap illustrating the spatial distribution and case intensity of reported meningitis cases across the Regional Director of Health Services (RDHS) divisions in Sri Lanka for the years 2023, 2024, and 2025 (Figure 2a–c).

The highest incidence of meningitis in Sri Lanka for 2023 and 2024 was concentrated in the Kurunegala, Gampaha, and Rathnapura RDHS divisions [21, 22]. In 2025, the districts of Kurunegala, Gampaha, and Galle documented a high incidence of cases, as shown in Figure 2 [19].

In May 2026, health authorities reported an increase in suspected viral meningitis outbreak among schoolchildren in the Deniyaya area in Matara [23]. Several affected students required hospitalization, and preventive measures, including temporary school closures and close epidemiological monitoring, were implemented to limit disease transmission [23]. Preliminary investigations conducted by health authorities suggested that the outbreak was associated with a viral infection potentially transmitted through contaminated water sources. According to the Government Medical Officers′ Association (GMOA), specialist teams from the Epidemiology Unit initiated field investigations to identify the source of infection, trace transmission patterns, and implement appropriate control and preventive measures in the affected area [24].

## 5. Types of Meningitis and Causative Organisms

Meningitis can be caused by various infectious agents, including bacteria, viruses, fungi, and parasites. Among these, bacterial meningitis remains a life‐threatening neurological condition worldwide, characterized by high morbidity and mortality and requiring immediate diagnosis and treatment [25]. In Sri Lanka, bacterial meningitis represents the most severe form of meningitis, affecting the CNS [8, 20].

Several bacterial species are known to cause meningitis, including *N. meningitidis*, *S. pneumoniae*, Hib, *Streptococcus agalactiae* (Group B *Streptococcus*), *Mycobacterium tuberculosis*, *E. coli*, *Listeria monocytogenes*, and *Klebsiella pneumoniae* [2, 26]. Prior to the introduction of the Hib vaccine into Sri Lanka′s National Immunization Program (NIP), Hib, Group B *Streptococcus*, and *S. pneumoniae* were common causative agents [20]. Additionally, documented clinical surveillance has reported *N. meningitidis*, *E. coli*, and Group D *Streptococcus* cases across local healthcare institutions [27].

*N. meningitidis* is responsible for meningococcal disease, which primarily presents as meningococcal meningitis or meningococcal septicemia (meningococcemia) [22]. Meningococcal septicemia, though less common, is a severe and rapidly progressive condition with high mortality due to systemic complications [28]. The pathogen is classified into at least 13 serogroups based on capsular polysaccharide, with Serogroups A, B, C, W135, X, and Y responsible for the majority of invasive disease globally [29].

Systematic surveillance continues to depend on passive surveillance. In Sri Lanka, severe forms of invasive meningococcal disease have been described in independent clinical investigations. For example, case series in a tertiary hospital setting have described patients with acute meningococcal septicemia with classical purpura fulminans, demonstrating rapid endotoxin release causing severe disseminated intravascular coagulation (DIC) and peripheral gangrene in adult patients [30]. Moreover, focused surveillance of military and community groups has revealed isolated outbreaks of particular serogroups, which demonstrates that whereas overall transmission rates are low, the lack of routine vaccination renders institutions extremely susceptible to acute meningococcemia [27].

Before the Hib vaccine was introduced in January 2008, Hib was the most common cause of bacterial meningitis in Sri Lanka [10]. Tuberculous meningitis, caused by *M. tuberculosis*, also remains a significant infectious disease with severe consequences, particularly in resource‐limited countries like Sri Lanka [31]. Additionally, a case reported in Sri Lanka described community‐acquired *K. pneumoniae* meningitis presenting with typical features of acute bacterial meningitis in a diabetic patient, highlighting the significant clinical challenges posed by hypervirulent strains in the country [32].

Viral meningitis is the most common type of meningitis, often referred to as aseptic meningitis, and can be caused by a wide range of viral pathogens. Enteroviruses are the most common causative agents globally. Other notable viruses include herpes simplex viruses (HSV‐1 and HSV‐2), varicella‐zoster virus (VZV), HIV, arboviruses, and Epstein–Barr virus (EBV) [33]. In populations with incomplete vaccination coverage, the mumps virus remains a significant cause, whereas cytomegalovirus (CMV) is frequently reported in immunocompromised hosts [34, 35]. In Sri Lanka, clinical investigations have identified CMV, enteroviruses, HSV‐1, HSV‐2, and VZV among the etiological agents of viral meningitis, with enteroviruses firmly established as the predominant cause of aseptic viral meningitis in the country [36]. Epidemiological surveillance indicates that enteroviral meningitis often exhibits seasonal patterns which correlate with rainy seasons and local water contamination outbreaks, such as the acute outbreak observed among schoolchildren in Deniyaya in May 2026 [23, 24]. However, because many regional hospitals lack routine access to multiplex PCR panels, a significant portion of viral etiologies remain underreported or broadly classified as aseptic without specific pathogen identification.

A wide range of fungal species are capable of causing meningitis. These include yeast species such as *Cryptococcus neoformans*, *Candida* spp., *Cryptococcus* spp., and *Trichosporon* spp.; filamentous fungi like *Aspergillus* spp., *Penicillium* spp., and *Pseudallescheria* spp.; dimorphic fungi such as *Blastomyces dermatitidis*, *Histoplasma capsulatum*, *Coccidioides* spp., and *Sporothrix* spp.; and dematiaceous fungi such as *Cladophialophora bantiana* and *Exophiala dermatitidis* [37]. Among these, *Cryptococcus* spp. stands out as the predominant cause of fungal meningitis globally [37, 38].

In Sri Lanka, cases of both *Aspergillus* meningitis and cryptococcal meningitis have been reported. *Aspergillus* meningitis was notably documented during a 2005 outbreak linked to spinal anesthesia [39]. Cryptococcal meningitis has been described in several individual case reports [40, 41].

Most parasitic meningitis cases present as eosinophilic meningitis (EM), a rare form of meningitis. *Angiostrongylus* spp. are recognized as the predominant global cause [42]. Another notable parasitic contributor to the condition is *Gnathostoma* spp. [43]. EM can also arise from several parasitic diseases, such as toxocariasis, schistosomiasis, hydatidosis, and strongyloidiasis [43, 44].

## 6. Clinical Presentation and Risk Factors

Meningitis presents with a set of well‐recognized clinical features worldwide, though the exact manifestations differ depending on the patient′s age, the causative organisms, and individual host factors. Early warning signs commonly include fever, headache, neck stiffness, photophobia, vomiting, and changes in mental status [45]. In infants and very young children, the presentation is often less specific, with symptoms such as irritability, poor feeding, lethargy, or a bulging fontanelle [46]. The clinical presentation of meningitis in Sri Lanka reflects both global patterns and pathogen‐specific features. However, locally published data remains limited. Most hospital‐based studies from Sri Lanka describe fever, headache, neck stiffness, purpuric skin rashes, altered consciousness, and seizures as common presenting symptoms, though the frequency of these findings is often not clearly documented [30, 47].

Age is a major risk factor for meningitis, particularly in the prevaccine era. Studies from Colombo, Sri Lanka, indicated that Hib meningitis was especially common among children younger than 5 years, highlighting how underdeveloped immune systems in this age group increase vulnerability [10]. Bacterial meningitis is a severe infection in children, with sensorineural hearing loss being a common complication. If hearing impairment is not detected early, affected children may experience subsequent speech and learning difficulties, as well as challenges in social development. In some cases, these issues can be misattributed to conditions such as intellectual disability, autism spectrum disorder, or childhood schizophrenia [9].

One of the important aspects of the long‐term public health burden of meningitis is the development of permanent neurological sequelae. Survivors of acute bacterial meningitis frequently suffer from severe postinfectious disabilities including sensorineural hearing loss, cognitive impairment, focal neurological deficits, and secondary epilepsy [48]. Hearing impairment has been highlighted as a major long‐term complication that needs to be monitored early through auditory‐evoked responses in local pediatric data from Colombo, and there is a deficit in systematic long‐term surveillance of neurodisabilities among all age groups in Sri Lanka [9]. Recent comprehensive epidemiological studies have shown a remarkably high burden of postmeningitis sequelae worldwide. For instance, a large multicenter study by Mohanty et al. [49] showed that a significant percentage of bacterial meningitis survivors had persistent neurological deficits at discharge and long‐term follow‐up, underscoring that survival itself does not adequately reflect clinical success. Sri Lanka lacks a standardized national registry to monitor these postdischarge neurological disabilities, hiding the true socioeconomic and rehabilitative burden of the disease.

Although clear gaps exist in the Sri Lankan literature, the available studies are typically limited by small sample sizes, single‐center designs, or a focus on isolated outbreaks. Broader information on clinical features, vaccination history, socioeconomic influences, coexisting illnesses, and delay in seeking care remains scarce. This gap underlines the need for large, multicenter epidemiological studies to provide a more complete understanding of meningitis presentation and risk factors across diverse regions and age groups in Sri Lanka.

## 7. Diagnostic Approaches in Sri Lanka

Accurate diagnosis is critical in meningitis management. In clinical practice, cerebrospinal fluid (CSF) analysis is the baseline diagnostic approach for suspected meningitis cases [20]. Blood investigations serve as additional supportive methods [47]. Culture remains the gold standard for identifying meningococcal meningitis [28]. In patients with clinically suspected bacterial CNS infections, CSF and blood cultures are routinely performed. However, conventional culture techniques, while highly specific, have relatively low sensitivity for etiological diagnosis. CSF gram stain, cytology, and biochemistry can provide early clues to the cause of meningitis and help distinguish between viral, bacterial, and fungal infections. Nevertheless, these tests are not always reliable due to their limited sensitivity [28]. In Sri Lankan studies, latex agglutination tests have been used to detect bacterial meningitis pathogens [10].

Nucleic acid amplification tests (NAATs), such as PCR, offer high sensitivity and specificity and are important for diagnosing both viral and bacterial meningitis. However, routine molecular‐based diagnostic facilities are not available in all Sri Lankan hospitals [11]. Multiplex PCR is recognized as the most effective molecular technique for identifying the causative pathogens rapidly, bypassing the delays associated with traditional cell culture [11]. Rapid antigen tests, such as immunochromatographic tests (ICTs), provide quick results for bacterial meningitis but are less sensitive and specific compared with PCR and culture, especially if antibiotics have been administered prior to testing [29]. Magnetic resonance imaging (MRI) of the brain is recommended as a key investigation in complicated meningitis cases and serves as an essential tool for accurate diagnosis [31].

## 8. Vaccination and National Immunity Gaps

Vaccination plays a crucial role in the prevention of bacterial meningitis, which was once common when Hib was recognized as the dominant cause of bacterial meningitis in Sri Lankan children [10]. However, in January 2008, the country successfully introduced the Hib vaccine in its NIP through the pentavalent formulation [50] which was predicted to reduce the incidence of bacterial meningitis by nearly 50% [10]. However, substantial systemic immunity gaps persist for other key pathogens.

There is a major vulnerability with *S. pneumoniae*. While Hib coverage is widespread, the inclusion of the PCV, in particular formulations like PCV7, continues to be absent from the routine national schedule despite its clear necessity to protect children from common invasive serotypes [51]. More drastically, despite vaccination remaining the most effective strategy for preventing meningococcal disease, these vaccines are completely excluded from the routine NIP [19]. Conjugate options targeting major virulent serogroups are available privately, including the tetravalent MenACWY formulation and the Serogroup B‐specific MenB vaccine [29]. However, their omission from the universal public schedule creates an absolute, unmitigated immunity gap against Serogroups A, C, W, Y, and B [52]. This lack of structural protection is by far worsened by the reliance on passive epidemiological surveillance and regional differences in vaccine uptake [19]. Consequently, highly vulnerable populations including specifically pediatric cohorts under 5 years of age and dense regional school clusters remain structurally unprotected against sudden shifts in circulating meningococcal serogroups and pneumococcal serotypes, transforming theoretical immunity gaps into localized public health vulnerabilities.

To overcome the limitations of the current preventive protocols, it is important to move to new vaccination strategies for long‐term control. Traditional polysaccharide–protein conjugate vaccines have been successful in targeting specific serogroups, yet future directions must utilize next‐generation formulations to overcome serotype replacement and localized strain gaps [53]. This includes clinical assessment and eventual deployment of higher valent PCVs (such as PCV15, PCV20, or newly engineered 24‐valent formulations) to widen coverage beyond historical baselines [54]. Furthermore, the evolution of universal protein‐based vaccines designed primarily against conserved surface antigens from multiple pneumococcal and meningococcal strains, rather than volatile capsular polysaccharides, represents a revolutionary approach independent of circulating serotype shifts [55]. Investigating maternal immunization routes against Group B *Streptococcus* also provides a critical, preventive opportunity to extend passive immunity to newborns during their most vulnerable neonatal window [56].

## 9. Current Management in Sri Lanka

Management of meningococcal meningitis focuses on prevention through vaccination and, in certain situations, chemoprophylaxis. Once bacterial meningitis is clinically suspected and a lumbar puncture has been performed, prompt initiation of antimicrobial therapy is essential to improve outcomes [20].

Current treatment strategies in Sri Lanka include the use of empirical intravenous antibiotics such as ceftriaxone or cefotaxime for suspected bacterial meningitis, whereas additional antibiotics like vancomycin or ampicillin may be added in high‐risk patients depending on age, immune status, and suspected pathogens [27, 29]. Additionally, individuals traveling to regions experiencing meningococcal outbreaks are strongly advised to receive vaccination as a precautionary measure. Furthermore, preventive and control measures such as infection surveillance, contact tracing, public health education, and outbreak investigations are increasingly emphasized in Sri Lanka to reduce disease transmission and improve patient outcomes [24, 57].

## 10. Gap and Future Directions

While notable progress has been made in reducing the burden of meningitis in Sri Lanka, several critical gaps remain in the country′s control efforts, mainly in immunization coverage, serogroup surveillance, early detection, and access to advanced diagnostics. A significant immunity gap persists, as a meningococcal vaccine is still not included in the national immunization schedule [29]. Furthermore, there is limited understanding of the coverage provided by current vaccines against all circulating serogroups. A critical step in introducing vaccines in resource‐limited settings is to evaluate the economic feasibility of altering the national immunization schedule [58]. A health economics report from Sri Lanka not only identified the preventive value of the meningococcal vaccine but also highlighted the need for national policy implementation to carefully consider broader financial constraints against local disease burden metrics to assess true feasibility [58]. The emergence of multidrug‐resistant meningococcal strains underscores the need not only to expand vaccination for disease prevention but also to curb antimicrobial resistance [27].

The dynamics of disease burden in Sri Lanka show clear differences with other South Asian countries in the analysis of regional epidemiological trends [59, 60]. In continental areas like India, the epidemiology of bacterial meningitis is characterized by a significant burden of *S. pneumoniae* and Hib [61], with occasional epidemics of *N. meningitidis* Serogroups A and C, driven largely by dense population and different regional health infrastructures [62]. In contrast, Sri Lanka′s unique island geography, high baseline literacy, and centralized public healthcare delivery have yielded a more controlled, lower overall incidence rate (1000–1500 annual cases) but a highly concentrated operational risk regarding localized outbreaks and diagnostic delays [59, 62]. However, whereas India has progressively introduced PCV into its universal immunization framework to aggressively combat pneumococcal burden [63, 64], Sri Lanka′s absolute reliance on a private market model for both PCV and meningococcal vaccines creates an unmitigated structural inequity [18].

Early diagnosis and surveillance capabilities are necessary to prevent the further transmission of meningitis [47]. Serological tests can help identify parasitic causes of meningitis, but these diagnostic tests are currently not widely available in Sri Lanka, limiting early and accurate diagnosis of parasitic infections [43]. The lack of laboratory facilities to identify *N. meningitidis* serogroups hampers proper surveillance and delays outbreak detection [28]. In low‐ and lower middle‐income countries (LMICs), low pathogen isolation rates are common due to inadequate diagnostic infrastructure, a challenge also observed in Sri Lanka [65]. Despite the availability of standard diagnostic procedures, several preanalytical and logistical barriers hinder accurate meningitis diagnosis. These include the administration of antibiotics prior to CSF collection, delays in sample acquisition and transport, and the collection of insufficient CSF volume. Such practices significantly reduce test sensitivity and often yield inconclusive findings [20]. These operational gaps show the urgent need to standardize and improve early detection methods, optimize sample collection and transport systems, and enforce stringent preanalytical procedures to enhance the reliability of laboratory diagnostics.

A further critical challenge is the limited access to rapid, highly sensitive diagnostic tests. While molecular techniques like multiplex PCR demonstrate superior sensitivity and accuracy in pathogen identification [11], their high cost remains a prohibitive barrier to routine implementation in many Sri Lankan healthcare facilities. Future directions should focus on early diagnosis methods like rapid diagnostic tests and integrating advanced molecular diagnostics like PCR and sequencing into routine hospital practice to address local diagnostic infrastructure gaps [6]. Furthermore, expanding serotype surveillance, strengthening vaccination introduction in the NIP, and implementing decentralized rapid diagnostic networks are critical to building a more responsive, evidence‐based meningitis control framework aligned with global health targets [5, 66].

We need an integrated framework that is based on diverse primary clinical data, rather than traditional reliance on general secondary reviews. When comparing localized pediatric auditory tracking [9] with multicenter international disability data [49], it becomes apparent that survival metrics hide a substantial hidden burden of postmeningitis sequelae. Furthermore, the trade‐offs between the updates of the molecular carriage [67] and regional frameworks in South Asia [17, 63] emphasize that the immunity gaps of Sri Lanka cannot be addressed by static policies. Future strategies must rapidly incorporate next‐generation universal protein vaccines and decentralized public PCR networks to convert the nation′s public health framework into an evidence‐based, scientifically independent structure.

To execute this, prioritizing interventions in Sri Lanka requires moving away from broad extrapolations and establishing a tiered, cost‐effective framework: first, upgrading public hospital diagnostic infrastructure with affordable multiplex PCR panels to map true local serotype distribution and second, leveraging this national baseline data to conduct localized catch‐up vaccination campaigns in highly dense regional clusters [18, 62].

## 11. Conclusion

Meningitis remains a significant public health concern in Sri Lanka, despite progress in vaccination coverage and enhanced disease surveillance. The reduction in meningitis cases from 2020 to 2022 can be attributed to the COVID‐19 pandemic. Public health protocols during this time effectively limited the transmission of multiple diseases. Current evidence demonstrates that bacterial and viral pathogens are predominant etiological agents for meningitis. Critical challenges persist in early diagnosis, laboratory confirmation, access to advanced molecular testing, and the implementation of a comprehensive national immunization strategy. However, important immunity gaps persist due to limited pneumococcal serotype coverage, the absence of routine meningococcal vaccination, and variable immunization coverage by region, as observed during specific catch‐up campaigns or localized surveillance assessments [19, 67]. Hospital‐based studies highlight delays in clinical presentation, difficulties in pathogen identification, and a lack of multicenter epidemiological data, showing the need for more robust and representative research. The integration of advanced molecular diagnostics, such as multiplex PCR, into routine hospital practice is crucial for rapid and accurate etiological identification, which is essential for guiding timely and appropriate therapy.

Strengthening diagnostic capacity, improving surveillance and reporting accuracy, introducing advanced molecular methods in clinical settings, expanding access to rapid diagnostics, and introducing broader spectrum vaccines are essential to reducing meningitis‐associated mortality and long‐term disability. Future priorities should focus on enhancing laboratory‐based surveillance, evaluating the feasibility of enhancing meningococcal vaccines into the national immunization schedule, and aligning local public health initiatives with the global targets of the WHO Defeating Meningitis by 2030 global roadmap. By systematically addressing these gaps in immunity, diagnostics, and surveillance, Sri Lanka can make meaningful progress toward controlling and eventually eliminating meningitis as a major public health threat.

## Author Contributions

T.H. and D.S. contributed equally to this work. T.H. and D.S. prepared the first draft of the manuscript. S.M., S.A.E., and B.D. guided the preparation of the manuscript and critically reviewed and revised the manuscript.

## Funding

No funding was received for this manuscript.

## Disclosure

All authors read and approved the final manuscript.

## Conflicts of Interest

The authors declare no conflicts of interest.

## Data Availability

Data sharing is not applicable to this article as no datasets were generated or analyzed during the current study.
